# Quantitative performance of ^124^I PET/MR of neck lesions in thyroid cancer patients using ^124^I PET/CT as reference

**DOI:** 10.1186/s40658-018-0214-y

**Published:** 2018-07-19

**Authors:** Walter Jentzen, Jinda Phaosricharoen, Benedikt Gomez, Philipp Hetkamp, Vanessa Stebner, Ina Binse, Sonja Kinner, Ken Herrmann, Amir Sabet, James Nagarajah

**Affiliations:** 1 Department of Nuclear Medicine, University of Duisburg-Essen, Hufelandstrasse 55, D-45122 Essen, Germany; 2 Radboudumc, Department of Radiology and Nuclear Medicine, Nijmegen, The Netherlands; 3 Department of Radiology, University of Duisburg-Essen, Essen, Germany; 4 Department of Nuclear Medicine, University Hospital Frankfurt, Frankfurt, Germany

**Keywords:** PET/MR, PET/CT, Iodine-124, Differentiated thyroid cancer

## Abstract

**Background:**

In patients with differentiated thyroid cancer (DTC), serial ^124^I PET/CT imaging is, for instance, used to assess the absorbed (radiation) dose to lesions. Frequently, the lesions are located in the neck and they are close to or surrounded by different tissue types. In contrast to PET/CT, MR-based attenuation correction in PET/MR may be therefore challenging in the neck region. The aim of this retrospective study was to assess the quantitative performance of ^124^I PET/MRI of neck lesions by comparing the MR-based and CT-based ^124^I activity concentrations (ACs). Sixteen DTC patients underwent PET/CT scans at 24 and 120 h after administration of about 25 MBq ^124^I. Approximately 1 h before or after PET/CT examination, each patient additionally received a 24-h PET/MR scan and sometimes a 120-h PET/MR scan. PET images were reconstructed using the respective attenuation correction approach. Appropriate reconstruction parameters and corrections were used to harmonize the reconstructed PET images to provide, for instance, similar spatial resolution. For each lesion, two types of ACs were ascertained: the maximum AC (max-AC) and an average AC (avg-AC). The avg-AC is the average activity concentration obtained within a spherical volume of interest with a diameter of 7 mm, equaling the PET scanner resolution. For each type of AC, the percentage AC difference between MR-based and CT-based ACs was determined and Lin’s concordance correlation analysis was applied. Quantitative performance was considered acceptable if the standard deviation was ± 25% (precision), and the mean value was within ± 10% (accuracy).

**Results:**

The avg-ACs (max-ACs within parentheses) of 74 lesions ranged from 0.20 (0.33) to 657 (733) kBq/mL. Excluding two lesions with ACs of approximately 1 kBq/mL, the mean (median) ± standard deviation (range) was − 4% (− 5%) ± 14% (− 28 to 29%) for the avg-AC and − 9% (− 11%) ± 14% (− 33 to 33%) for the max-AC. Lin’s concordance correlation coefficients were ≥ 0.97, indicating substantial AC agreement.

**Conclusions:**

Quantification of lesions in the neck region using ^124^I PET/MR showed acceptable quantitation performance to ^124^I PET/CT for AC above 1 kBq/mL. The PET/MRI-based ^124^I ACs in the neck region can be therefore reliably used in pre-therapy dosimetry planning.

## Background

Positron emission tomography (PET) in combination with magnetic resonance imaging (MR) is becoming an emerging tool for cancer imaging [1, 2]. Although a high number of PET/MR studies have been published in the last years, no clear clinical indications have been established yet. This is mainly associated with the lack of proper prospective studies and with misquantification of PET activity concentrations (ACs) of target lesions requiring proper attenuation correction. Despite many different approaches, a widely accepted robust attenuation correction technique for PET/MR remains challenging [3].

Moreover, some clinical studies published so far indicated, on average, discrepant results regarding the ACs or, equivalently, the standard uptake values of corresponding lesions in PET/MR compared to PET/computer tomography (CT) [4, 5]. Those studies assumed that the observed differences are mainly due to radiopharmacokinetic properties of the PET tracers used, mainly bound to the short-living radionuclides ^18^F and ^68^Ga. In addition, those authors probably did not “harmonize” PET imaging reconstruction parameters such as voxel size, smoothing level, and the number of effective iterations.

To reduce the influence of the radiopharmacokinetics, we analyzed the ACs of ^124^I in thyroid cancer patients. ^124^I has slower kinetics in thyroid tissue or metastases compared to the kinetics of ^18^F-FDG-accumulating tissues or metastases, and most importantly, ^124^I exhibits a notably longer physical half-life of approximately 4 days. Specifically, ^124^I PET/CT has been used to perform lesion dosimetry prior to radioiodine treatment in patients with differentiated thyroid cancer (DTC) [6–9]. A reliable quantification is crucial for (radiation) absorbed dose estimations in lesions. Frequently, the lesions in DTC patients are located in the neck area and close to or surrounded by different types of tissues like trachea, muscles, salivary glands, and bones, which contribute differently to the attenuation correction; therefore, MR-based attenuation correction in PET/MR may be impaired. Additionally, in the present study, PET reconstruction parameters were harmonized for both scanner systems for a more reliable AC comparison between PET/MR and PET/CT.

The aim of this retrospective study was to compare the MR-based ^124^I ACs of lesions located in the neck area in thyroid cancer patients with the respective CT-based ^124^I ACs serving as reference standard.

## Methods

### Patients and lesions

The local ethics research committee approved the study. The study included 16 high-risk patients (7 women, 9 men) prior to their first radioiodine therapy. All patients underwent total thyroidectomy and had histologically confirmed advanced DTC (papillary in 13 and follicular in 3 cases). Mean ± standard deviation (SD) age was 54 ± 21 years. The thyroid-stimulating hormone (TSH) stimulation was achieved by withdrawal of thyroid hormone in 15 cases for about 4 weeks and by injection of recombinant human TSH (Thyrogen, Genzyme, GmbH, Frankfurt, Germany) in 1 case; the mean ± SD of TSH level before imaging was 74.2 ± 57.4 IU/mL. All patients followed a low-iodine diet for 4 weeks prior to ^124^I PET examination, and iodine contamination was excluded by urine testing. The patients were administered an activity of approximately 25 MBq ^124^I.

As we acquired one-bed PET/MR scans of the neck, we included only lesions located within this region in this quantitative performance study. The lesions were clearly conspicuous and unambiguously identified on both PET/CT and PET/MR images. Moreover, all lesions were classified as either lymph node metastasis or thyroid remnant tissue using localization criteria. In addition, to study the effect of possible missegmentation of soft tissue as, for instance, “air” or “lung tissue” in the attenuation correction approach, and the lesions were categorized in two groups in terms of their distance measurement from the trachea surface, that is, adjacent (distance ≤ 5 mm) or distant (distance > 5 mm).

### PET/CT imaging

Imaging was performed on two PET/CT scanners, Biograph mCT PET/CT and Biograph Duo PET/CT (Siemens Healthcare, Erlangen, Germany). The whole-body PET/CT scans were conducted in the context of ^124^I lesion dosimetry. In particular, each patient received either serial PET/CT scans on the Biograph mCT PET/CT system or on the Biograph Duo PET/CT system. The PET/CT scans were acquired approximately at 24 and 120 h. This two-point protocol was used as it is a reliable simplification of a comprehensive five-point protocol to estimate the time-integrated activity (TIA) coefficients (erstwhile known as residence times) [10]. The examinations included PET/CT scans from head to thigh using 5–8 bed positions. During PET/CT acquisition, the patient’s arms were positioned above the head. CT imaging was performed without iodine-containing contrast agent to avoid interference with radioiodine uptake. For both PET/CT systems, the scans started with a CT in low-dose technique. Standard corrections for random coincidence, scatter, and dead time were performed. Images were corrected for attenuation with a CT-based attenuation correction method. The two scanners were cross-calibrated with ^18^F using a dose calibrator Isomed 2100 (MED Nuklear-Medizintechnik, Dresden GmbH, Germany).

Image acquisition and image reconstruction differed among the PET/CT scans (detailed information available in Table 1. For the Biograph mCT PET/CT system, a total of 8 PET images were analyzed, each was acquired with an emission time of 2 min per bed position. A three-dimensional (3D) ordinary Poisson ordered-subset expectation maximization (OP-OSEM) algorithm was used. Sinogram-based correction of prompt-gamma coincidences was performed for ^124^I using the standard manufacturer’s reconstruction software [11]. For lesion dosimetry, the image reconstruction parameters were 6 iterations and 12 subsets and a 3D Gaussian smoothing filter of 4 mm was applied. The estimated reconstructed PET spatial resolution (expressed as the full width at half maximum) was 6.8 mm. Six images were reconstructed with a reconstructed voxel size of 1.5 × 1.5 × 1.5 mm^3^ (2.1 × 2.1 × 2.4 mm^3^). The respective CT images were reconstructed using the standard reconstruction kernel B40s with a voxel size of 1.0 × 1.0 × 2.4 mm^3^ (1.5 × 1.5 × 1.50 mm^3^).

**Table 1:** Overview of a number of images and patients/lesions as well as PET image reconstruction parameters before image harmonization for the different PET scanners

System^a^	Images	Number of patient	Early and/or late images	Number of lesions	Emission time (min)^b^	Iteration/Subs.	Voxel size (mm^3^)	3D Gaussian filter (mm)	Resolution (mm)^c^
Duo	10	5	24 h + 120 h	24	3.5	4/16	1.73 × 1.73 × 2.43	0	6.6
	7	7	24 h	20	3.5	4/16	1.53 × 1.53 × 2.43	0	6.6
mCT	2	1	24 h + 120 h	8	2	6/12	2.04 × 2.04 × 2.03	4	6.8
	6	3	24 h + 120 h	22	2	6/12	1.45 × 1.45 × 1.50	4	6.8
mMR	12	6	24 h + 120 h	32	8	3/21	2.09 × 2.09 × 2.03	5	7.0
	6	3	24 h + 120 h	22	8	3/21	2.09 × 2.09 × 2.03	4	6.3
	5	5	24 h	16	8	3/21	1.74 × 1.74 × 2.03	4	6.3
	2	2	24 h	4	8	3/21	2.09 × 2.09 × 2.03	4	6.3

^a^Biograph Duo PET/CT, Biograph mCT PET/CT, and Biograph mMR PET/MR

^b^Emission time per bed

^c^Estimated reconstructed PET spatial resolution

For the Biograph Duo PET/CT, a total of 18 PET images were analyzed (detailed information available in Table 1). The emission time was 3.5 min per bed position. After Fourier-rebinning, an attenuation-weighted ordered-subset expectation maximization (AW-OSEM) algorithm was used. Sinogram-based correction of prompt-gamma coincidences was not available for this system. The standard image reconstruction parameters for lesion dosimetry were 4 iterations and 16 subsets. No Gaussian smoothing filter was applied, resulting in a PET spatial resolution of 6.6 mm. Ten images (8 images within parenthesis) were reconstructed with a reconstructed voxel size of 1.7 × 1.7 × 2.4 mm^3^ (1.5 × 1.5 × 2.4 mm^3^). The CT images were reconstructed using the standard reconstruction kernel B40s with a voxel size of 1.0 × 1.0 × 2.4 mm^3^.

### PET/MR imaging

In the context of detecting and categorizing cervical iodine-positive lesions, the Biograph mMR PET/MR (Magnetom Biograph mMR; Siemens Healthcare, Erlangen, Germany) was performed 24 h after ^124^I administration (16 images), and a 120-h PET/MR was additionally acquired in 10 cases, resulting in a total of 26 images. The one-bed scans of the neck were acquired at approximately 1 h before or after the PET/CT scans. The patient’s arms were positioned alongside of the body. MR was performed simultaneously before and after administration of contrast medium (0.2 mL/kg body weight Dotarem; Guerbet GmbH, Sulzbach, Germany) using a head-and-neck coil. Attenuation correction was based on an automatically generated four-compartment model attenuation map (μ-map) derived from a two-point T1-W Dixon VIBE (volumetric interpolated breath-hold examination) sequence [12]. Standard corrections for random coincidence, scatter, and dead time were performed. Sinogram-based correction of prompt-gamma coincidences was performed for ^124^I [11] (detailed image reconstruction parameters are given in Table 1). Specifically, PET image was reconstructed using an OP-OSEM algorithm (3 iterations, 21 subsets). Twelve images had a voxel size of 2.1 × 2.1 × 2.0 mm^3^ and were smoothed with a 5-mm 3D Gaussian filter, resulting in an estimated spatial resolution of 7.0 mm. Nine images (5 images within parenthesis) were reconstructed with a reconstructed voxel size of 2.1 × 2.1 × 2.0 mm^3^ (1.7 × 1.7 × 2.0 mm^3^); the Gaussian smoothing filter was 4 mm for the 14 images, resulting in an estimated spatial resolution of 6.3 mm. The MR images had a voxel size identical with the respective PET images. The PET/MR scanner was cross-calibrated with ^18^F using the same dose calibrator as for the PET/CT scanners.

### Image harmonization and prompt-gamma coincidence scaling for improving quantitative comparability

To improve quantitative comparability in this retrospective study, two corrections were applied to match the different PET systems. First, as the PET images were reconstructed with different image reconstruction parameters (see Table 1), the reconstructed images were largely harmonized using reconstruction parameters that revealed almost similar PET spatial resolution of 7.0 mm (by selecting an appropriate 3D Gaussian filter for each system) and equivalent voxels size of 2.1 × 2.1 × 2.4 mm^3^ (by means of trilinear interpolation). This correction is termed image harmonization. Note that the number of iterations and subsets was unaltered with respect to the non-harmonized standard reconstructions as effective iterations were similar, that is, 64 (4 × 16), 72 (6 × 12), and 63 (3 × 21) for the Biograph Duo, Biograph mCT, and Biograph mMR, respectively. Of note, the different emission times among the PET scanners, resulting in different signal-to-noise ratios, could not be corrected for.

Second, ^124^I is a non-pure positron emitting radionuclide and exhibits a complicated decay scheme [13]. Specifically, approximately 12% of ^124^I decay are associated with a 605-keV prompt-gamma emission occurring subsequently with the emission of a positron. As its energy falls within the PET energy window and the prompt gammas correlate in time with the annihilation photons, prompt-gamma coincidences (PGCs) are produced. Thus, the quantification is impaired by PGCs [13] and its level of impairment is scanner-dependent. Consequently, algorithms are necessary to correct for PGCs. Comprehensive sphere phantom measurements with ^124^I under conditions typically observed in clinical thyroid cancer ^124^I PET imaging demonstrated that the imaged ^124^I AC is underestimated by 20% for the Biograph Duo PET/CT and by 10% for the Biograph mCT PET/CT [13] and Biograph mMR PET/MR [14], even though the two newest PET systems (mCT and mMR) included a PGC correction approach in their image reconstruction software. To improve quantitative comparability, a PGC scaling factor of 0.8 for the Biograph Duo PET/CT system and a factor of 0.9 for both the Biograph mCT PET/CT and the Biograph mMR PET/MR systems were applied. In the following, this correction is termed PGC scaling. Due to the minor effect of the magnetic field on the path of the positrons in PET/MR systems, particularly in clinical setting, this effect was not considered in this study.

### Quantitative performance metrics and the acceptance criteria

The quantitative performance of the PET/MR was assessed using the percentage deviation between MR-based and CT-based ^124^I ACs of each lesion and Lin’s concordance correlation (CC) coefficient. Two types of ACs and their percentage deviations were determined. In detail, a volume of interest (VOI) was drawn to determine the maximum AC (max-AC) and the average AC (avg-AC). The avg-AC is the average activity concentration obtained within a spherical VOI with a diameter of 7 mm, equaling the PET scanner resolutions [13]. The center of the spherical VOI was located at the voxel position of the maximum AC. Lin’s CC coefficient along with the two-sided lower and upper 95% confidence interval (CI) was used to assess the strength of AC agreement. We applied the commonly used McBride’s criteria [15], which designates Lin’s CC coefficient > 0.99 as almost perfect and 0.95 to 0.99 as substantial.

Quantitative performance was considered acceptable if the SD of the percentage differences was ± 25% (measure of precision) and the mean value was within ± 10% (measure of accuracy). Lin’s CC coefficient assesses both the measurement of precision (a Pearson correlation coefficient) and of accuracy (a bias correction factor, which measures the level of the deviation from a 45° line through the origin).

### Simulation-based assessment of the implication of varying performance levels on the precision of the lesion dosimetry

Using the MIRD concept, the TIAC is, inter alia, required to estimate absorbed dose to lesions. In recent thyroid dosimetry studies [6–9], we applied a two-point approach to estimate the TIACs for metastases and thyroid remnants: an early ^124^I AC (24 h) and a late ^124^I AC (120 h) to estimate the absorbed dose to lesions after projection to the therapeutic radioiodine nuclide ^131^I.

In the simulation, we estimated the uncertainty of the ^131^I TIAC contribution between 24 and 120 h or, in other words, the “area under the curve” of the (projected) ^131^I uptake curve between the two measured points. A mono-exponential function was used to estimate the ^131^I TIAC contribution (for a lesion of known volume). The simulation approach consisted of two parts. In the first part, the reference ^131^I TIAC was determined. More precisely, a reference ^124^I ACs at 24 h after administration was selected (first input parameter); the reference ^124^I ACs at 120 h was calculated using an assumed effective ^124^I half-life (second input parameter). In the second part, we applied identical relative uncertainties for the two ^124^I ACs as a first-order estimate. The AC uncertainty was expressed in a form of a percentage SD (third input parameter). Assuming a normal distribution for the ^124^I ACs allowed us to simulate the ^131^I TIAC distribution of the percentage difference from the reference ^131^I TIAC for different levels of relative SD of ^124^I AC for each ^124^I effective half-life. Similarly, the percentage SD of ^131^I TIAC derived from the simulated TIAC distribution served as relative uncertainty in the TIAC determination.

A plot was created from the simulation results to illustrate the effect of different percentage SDs of the ^124^I AC for a given effective ^124^I half-life on the percentage SD in the ^131^I TIAC contribution. Note that the percentage SD in the TIAC correlates with the percentage SD in absorbed dose.

### Statistics

The descriptive statistics included the mean, the median, the SD, the minimum, and the maximum, which are provided in the following form: mean (median) ± SD (minimum to maximum). Differences among the groups were evaluated by Mann-Whitney *U* test (non-parametric test). A significance level (*P* value) of less than 5% was considered statistically significant.

## Results

**Fig. 1:**
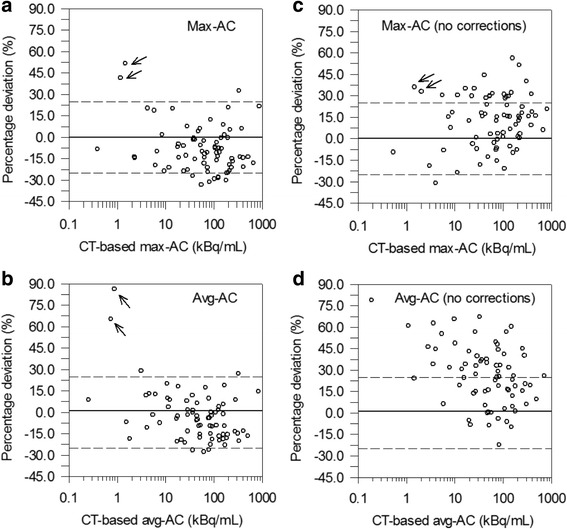
Percentage deviations between MR-based and CT-based ACs as a function of the CT-based max-AC and avg-AC with (**a**, **b**) and without corrections (**c**, **d**). Two outliers are marked with arrows. In panel **d**, the deviations of the outliers (98 and 142%) are beyond the axis scale and are not shown. Solid (dashed) lines represent the zero (± 25%) percentage deviations

**Fig. 2:**
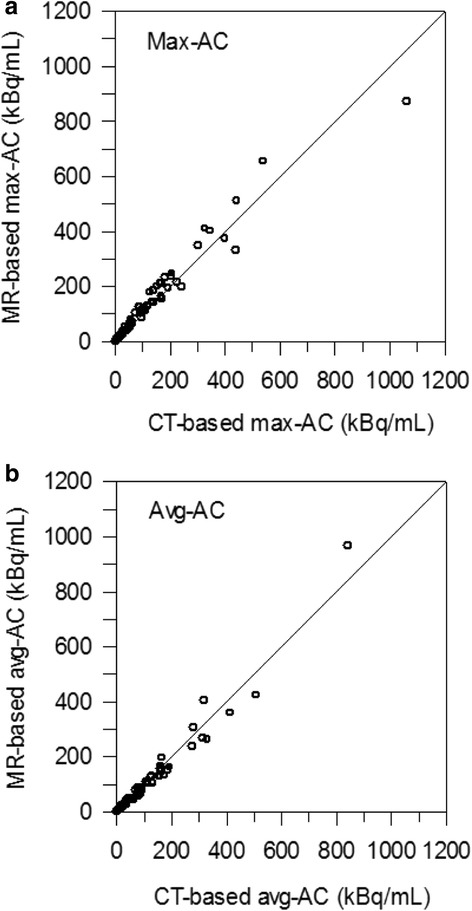
Lin’s CC plots of the max-AC (**a**) and avg-AC (**b**). Lines of identity (45° line) are shown by solid lines

The 16 patients had a total of 47 different lesions. Based on the lesion location within the neck area, 8 lymph node metastases and 39 thyroid remnants were univocally identified. Per patient, at least 1 lesion and up to 8 lesions were included. The statistics of the PET start time difference between PET/MR and PET/CT scans was 0.55 h (0.98) ± 1.24 h (− 2.1 to 2.5 h). For each type of AC, 74 ACs were determined; the avg-ACs (max-ACs within parentheses) ranged from 0.25 (0.39) to 842 (8723) kBq/mL. Figure 1a, b illustrates the percentage difference between PET/MR and PET/CT as a function of the CT-based ACs. As shown in Fig. 1, 2 thyroid remnants (marked with arrows) observed in the same patient had ACs of approximately 1 kBq/mL and exhibited a large deviation range (40 to 86%). Excluding these 2 lesions, the mean (median) ± SD (range) of the percentage AC difference was − 4% (− 5%) ± 14% (− 28 to 29%) for the avg-AC and–9% (− 11%) ± 14% (− 33 to 33%) for the max-AC (Table 2). Lin’s CC plots are shown in Fig. 2a, b for the max-AC and avg-AC, respectively. Lin’s CC coefficients were 0.98 (95% CI, 0.97 to 0.99) and 0.97 (95% CI, 0.95 to 0.98) for the avg-AC and max-AC, respectively, demonstrating substantial AC agreement. According to the acceptance criteria, ^124^I PET/MR showed acceptable quantitation performance to ^124^I PET/CT: the SD was within ± 25%, and the mean value was within ± 10%.

**Table 2:** Statistics of the percentage difference between MR-based and CT-based AC for different correction approaches (excluding two lesions considered as outliers)

Correction	Avg-AC	Max-AC
None	27% (26%) ± 21% (− 22 to 79%)	12% (15%) ± 18% (− 31 to 56%)
PGC scaling	18% (18%) ± 17% (− 23 to 60%)	11% (12%) ± 18% (− 33 to 51%)
Image harmonization	3% (1%) ± 19% (− 27 to 44%)	– 8% (− 10%) ± 16% (− 33 to 37%)
PGC scaling + image harmonization	– 4% (− 5%) ± 14% (− 28 to 29%)	– 9% (−11%) ± 14% (− 33 to 33%)

Statistics included the mean, the median, the SD, the minimum, and the maximum, which are provided in the following form: mean (median) ± SD (minimum to maximum). None means without any corrections. PGC scaling refers to only application of scaling factors. Image harmonization refers to only corrections pertaining to image reconstruction parameters. PGC scaling + image harmonization means application of both corrections, PGC scaling and image harmonization

For comparison purposes, we also determined the ACs without image harmonization and PGC scaling. In Table 2, an overview of the statistics of the percentage difference between MR-based and CT-based AC for different corrections (none, PGC scaling, image harmonization) are listed. The corresponding percentage difference without any corrections is shown in Fig. 1c, d. Likewise, excluding 2 lesions, the mean (median) ± SD (range) of the percentage AC difference was considerably larger, that is, 26% (27%) ± 21% (− 22 to 79%) for the avg-AC and 15% (12%) ± 18% (− 31 to 56%) for the max-AC. Without image harmonization and PGC scaling, quantitative performance was inacceptable primarily because of accuracy, that is, the mean MR-based AC was systematically overestimated by 26% for the avg-AC and 15% for the max-AC. Of note, the AC agreement primarily failed because of not performing image harmonization, whereas PGC scaling did not produce a dominant contribution in obtaining a better AC agreement.

**Fig. 3:**
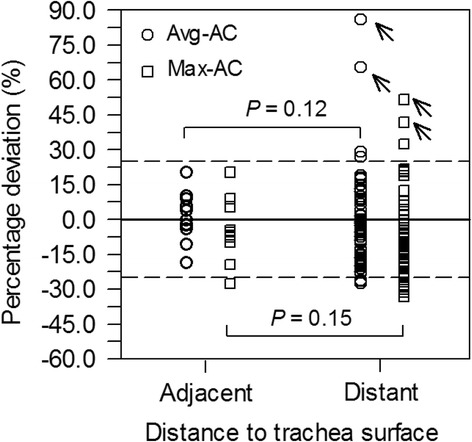
Percentage deviation between MR-based and CT-based ACs for lesions that are adjacent to (distance ≤ 5 mm) or distant from the trachea surface (distance > 5 mm). Outliers are marked with arrows. Solid (dashed) lines represent the zero (± 20%) percentage deviations

The percentage differences of lesions adjacent to (11 lesions) and distant from the trachea surface (63 lesions) are shown in Fig. 3. Excluding the 2 outliers, the mean (median) ± SD (range) of the percentage difference for the group of adjacent lesions was 1% (− 0%) ± 11% (− 19 to 20%) for the avg-AC and − 5% (− 7%) ± 13% (− 28 to 20%) for the max-AC. The statistics of the group of distant lesions was − 5% (− 7%) ± 14% (− 28 to 29%) for the avg-AC and − 10% (− 14%) ± 14% (− 33 to 32%) for the max-AC. No statistical significance in the AC differences for the respective type of AC was observed for the two lesion groups (*P* > 0.12).

**Fig. 4:**
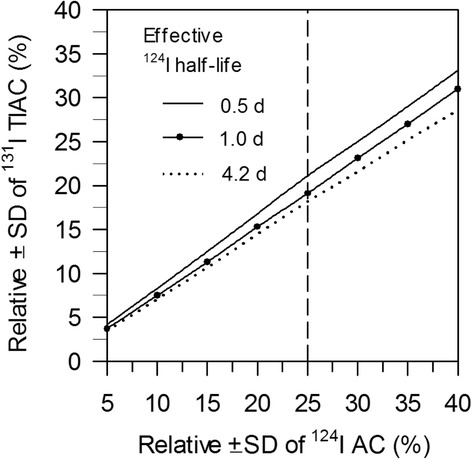
Simulated relative SDs of ^131^I TIAC contribution as a function of relative SD of ^124^I ACs. Dashed vertical line represents the accepted relative SD of ± 25% for ^124^I AC

Figure 4 illustrates the simulated relative uncertainties of the ^131^I TIAC contribution at different effective ^124^I half-lives as a function of the simulated relative ^124^I AC uncertainties. The relative SD of ^131^I TIAC remains below the ± 20% limit for relative SD of ^124^I AC of approximately 25%.

## Discussion

In this retrospective study, we compared the MR-based ^124^I ACs of lesions located in the neck area in thyroid cancer patients with the respective CT-based ^124^I ACs (as reference). We found that after image harmonization and PGC scaling, MR-based ^124^I ACs agreed well with the CT-based ^124^I ACs, that is, the mean percentage AC difference ± SD was− 4% ± 14% for the avg-ACs and − 9 ± 14% for max-ACs (Table 2 and Fig. 1) and the Lin’s CC plot exhibited substantial AC agreement (Lin’s CC coefficients were ≥ 0.97, Fig. 2).

Many authors [16, 17] raised concerns that image segmentation in MR-based attenuation correction with Dixon sequences is not reliable in regions, where different tissue types (soft tissue, bone, air) are in close proximity such as in the neck area. Even for lesions in the proximity of trachea, where the segmentation may influence the quantification, we could not observe any notable AC differences between lesions that are adjacent to (distance ≤ 5 mm) or distant from the trachea surface (distance > 5 mm) (Fig. 3), indicating that image segmentation did not contribute to a notable variation in ACs.

In addition, several authors [4, 5, 18] proposed pharmacokinetics as a source of the quantification differences. Heusch et al. [4] reported, on average, a significantly higher max-SUV and mean-SUV for PET/MRI compared to PET/CT of 13–21% (7.39 ± 6.7 vs. 6.09 ± 6.5 for max-SUV and 3.73 ± 2.9 vs. 3.3 ± 2.9 for mean-SUV; *P* < 0.001 each). A discrepant finding was observed by Wiesmüller et al. [5]. In contrast to Heusch et al. [4], they found, on average, lower values for max-SUV and mean-SUV for PET/MRI compared to PET/CT of 11–21% (13.91 ± 13.00 vs. 17.61 ± 15.50 for max-SUV and 5.6 ± 3.63 vs. 6.27 ± 3.88 for mean-SUV, each *P* < 0.01). Unfortunately, both studies did not consider image harmonization. Two factors may explain the discrepant findings: (i) pharmacokinetics of tracers with short half-lives in combination with a fast biokinetics and (ii) different PET image reconstruction parameters.

Both factors were considered in our study. The first factor was minimized using ^124^I PET images from thyroid cancer patients. Specifically, ^124^I has a long physical half-life of 4.2 days and the lesions exhibit a slow radioiodine biokinetics. In contrast, the importance of the second factor becomes obvious when considering the avg-ACs, for example. The avg-AC percentage difference after image harmonization was considerably reduced down to 3% (Table 2). Therefore, we conclude that image harmonization plays a crucial role.

Several issues have to be mentioned. Image harmonization could not be completely performed. The different emission times per bed position could not be harmonized as this is a retrospective study. Nevertheless, the AC differences are still acceptable in clinical settings. In addition, from Table 2, it can be concluded that PGC scaling appears to have a minor impact on AC quantification. Also, in our cohort, two outliers exhibited large percentage differences of about 45−90%. We suggest that this is mainly related to the low max-AC of approximately 1 kBq/mL (Fig. 1).

To understand the impact of the different levels of AC uncertainties on absorbed dose calculations, we performed a simulation study (see “Methods” section). As shown in Fig. 4, a SD of ± 15% in the ^124^I AC translated into an “absorbed dose distribution” of ± 12%, even a SD of ± 25% produced “absorbed dose distribution” of ± 20%. Thus, considering factors involved in the absorbed dose calculations, the authors deem that the ± 20% absorbed dose distribution is still acceptable. On the ground of these findings, we infer that protocols including PET/MR alone or in combination with PET/CT imaging for lesions located in the neck region are reliably applicable for dosimetry purposes. Finally, our results clearly substantiate that the “harmonization” of the scanning parameters is a critical step for a quantitative comparison between different PET systems.

## Conclusions

After image harmonization, quantification of lesions in the neck region using ^124^I PET/MR showed acceptable performance to ^124^I PET/CT. The MR-based ^124^I ACs in the neck region can be therefore reliably used in pre-therapy dosimetry planning.

## Abbreviations

AC: Activity concentration
AW-OSEM: Attenuation-weighted ordered-subset expectation maximization
CT: Computer tomography
DTC: Differentiated thyroid cancer
MR: Magnetic resonance tomography
OP-OSEM: Ordinary Poisson ordered-subset expectation maximization
PET: Positron emission tomography
PGCs: Prompt-gamma coincidences
TIA: Time-integrated activity
TSH: Thyroid-stimulating hormone
VIBE: Volumetric interpolated breath-hold examination
VOI: Volume of interest
